# A diverse group of halophilic bacteria exist in Lunsu, a natural salt water body of Himachal Pradesh, India

**DOI:** 10.1186/s40064-015-1028-1

**Published:** 2015-06-17

**Authors:** Sonika Gupta, Parul Sharma, Kamal Dev, Malay Srivastava, Anuradha Sourirajan

**Affiliations:** Faculty of Biotechnology, Shoolini University, Solan, 173212 Himachal Pradesh India

**Keywords:** Halophiles, Lunsu, Phylogenetic, RAPD, 16S rDNA, Blast

## Abstract

Five halophilic bacterial isolates namely SS1, SS2, SS3, SS5 and SS8 were isolated from soil sediments of Lunsu, a salty water body. All the bacterial isolates showed growth in LB medium containing up to 8.7% NaCl, pH 7–8 and at temperature range of 30–37°C. The bacterial isolates SS1 and SS3 require at least 3.8% NaCl for their growth, indicating their strict halophilic nature. Interestingly, bacterial isolates SS2, SS5 and SS8 but not SS1 and SS3 exhibited growth in medium supplemented with KCl. Accordingly, Na^+^ and K^+^ ions were detected at 1.39 and 0.0035%, respectively in Lunsu water. All the bacterial isolates were analyzed by random amplification of polymorphic DNA (RAPD) using four different random primers and produced PCR fragments ranging from 0.1 to 5 kb in size. Phylogenetic tree based on RAPD finger prints showed that SS1 and SS3 formed one group, while SS2 and SS5 formed the second group, whereas SS8 was out group. Sequence analysis of 16S rDNA identified SS1 and SS3 as *Halobacillus trueperi*, SS2 as *Shewanella algae,* SS5 as *Halomonas venusta,* and SS8 *as Marinomonas* sp. were deposited in GenBank with accession numbers of KM260166, KF751761, KF751760, KF751762 and KF751763, respectively. This is the first report on the presence of diverse halophilic bacteria in the foot hills of Himalayas.

**Electronic supplementary material:**

The online version of this article (doi:10.1186/s40064-015-1028-1) contains supplementary material, which is available to authorized users.

## Background

The presence of vast areas of saline water around the earth’s surface has provided favourable conditions for the evolution and emergence of salt loving organisms called halophiles. Halophilic microorganisms are primarily found in hypersaline environments and have been reported throughout the world (Oren 2002; Surve et al. 2012). Halophilic microbial communities have been well studied from hyper saline regions such as Great salt lake (USA), Dead Sea (Israel), Wadi Natrun lake (Egypt), lake Magadi (Kenya), soda lake (Antarctica) and Big Soda Lake and Mono Lake (California) (Litchfield and Gillevet 2002; DasSarma and DasSarma 2012). Hyper saline regions differ from each other in terms of salt concentration, chemical composition and geographical location, which determine the nature of inhabitant microorganisms. Halophiles are found in all the three domains of life such as Archaea (e.g., *Halobacterium* sp.), bacteria (e.g., *Halobacillus* sp.) and eukaryotes (e.g., green algae, *Dunaliella salina*, brine shrimp, *Artemia franciscana* and halophytic plant, *Atriplex halimus*) (DasSarma and DasSarma 2012). The cultured diversity of halophilic Archaea includes 47 genera and 165 species of the family *Halobacteriaceae* (Oren 2014). Hyper saline environments are predominantly inhabited by both extremely halophilic and halotolerant microorganisms such as *Halobacterium* sp., *Haloferax* sp., *Haloarcula* sp., *Halobacillus* sp., *Salinibacter ruber*, *Virgibacillus salarius*, *Bacillus* spp. *and Micrococcus luteus* (Paterekt and Smith 1985; Arahal et al. 1996; Anton et al. 2002; Solanki and Kothari 2012; Solomon and Viswalingam 2013). Halophilic microorganisms require at least 0.2 M salt for their growth and cannot grow in the absence of salt. On the other hand, halotolerant bacteria grow in the absence of salt as well as in the presence of relatively high salt concentrations (e.g., *Staphylococcusaureus* and *Vibrio* sp.) (Ara et al. 2013).

Halophiles possess the ability to balance the osmotic pressure of the environment. To do so, they either accumulate compatible solutes or uptake K^+^ ions in exchange of Na^+^ ions (Galinski 1993; Waditee et al. 2002; Roberts 2005; Ghasemi et al. 2011) or use both strategies (hybrid strategy) together (Saum et al. 2012; Hänelt and Müller 2013). Modification of membrane composition (Sakamoto and Murata 2002) and induction of general stress tolerance proteins are other means used by halophiles to cope up with salt stress (Hillmann et al. 2006; Kapardar et al. 2010).

There is a tremendous demand for halophilic bacteria due to their biotechnological importance as sources of halophilic enzymes, fermentation of soy and fish sauce, biological treatment of saline wastewater and production of β-carotene, compatible solutes, bioplastics and bio fuel (Oren 2010; Li and Yu 2012). In addition, halophilic microbes serve as an attractive source of salt-tolerance genes for the generation of salt resistant transgenic plants (Kapley et al. 1999; Gisbert et al. 2000; Klahn et al. 2009).

In India, halophilic micro flora have been reported from natural hyper saline habitats from the coastal regions of Maharashtra, Gujarat, Tamil Nadu, Goa and desert state of Rajasthan (Kokare et al. 2004, Dodia et al. 2006, Vijayanand et al. 2012, Surve et al. 2012, and Nigam et al. 2013). The bacteria reported from these locations (e.g., *Alkalibacillu*s sp. A1, *Virgibacillus* sp. V1 and *Actinopolyspora* sp. AH1) are halo-alkaliphilic in nature, and exhibit optimal growth in the presence of 5–20% NaCl and pH 8–10. In Himachal Pradesh, the hill state of India, there are two salt water bodies, one located in Gumma (Mandi district) and other in Lunsu (Kangra district). However, these saltern water bodies have not yet been explored for the presence of halophilic microbes. Therefore, we underlook this study to isolate and characterise the halophilic bacteria from Lunsu. Our results reveal the existence of strict halophilic as well as halotolerant bacteria in the sediment of Lunsu salt water stream.

## Methods

### Growth media

The media used in the present study were of microbiological grade, and procured from Himedia Labs, Mumbai. The halophilies were isolated and cultured in Luria broth (LB) or minimal M9 medium supplemented with NaCl (Sambrook et al. 2009).

### Sampling site and sample collection

Soil samples were collected in sterile 50 ml centrifuge tubes from the Lunsu water body situated in Kangra, Himachal Pradesh, India. The Lunsu water body is 10 km from Kangra railway station and located at the longitude of 76.1667362 and latitude of 32.0067136. The literal meaning of “luun” is salt in local language, and hence named Lunsu. Soil sample (~5 g) was collected from water body site and mixed with 5 ml of 2 × LB medium containing 3% NaCl and incubated at 30°C for 3 days, with intermittent mixing for the enrichment of halophilic bacteria. For estimation of salts in Lunsu water, water sample was collected in sterile 50 ml centrifuge tubes.

### Chemical analysis of water sample

The pH of water sample was recorded using digital pH meter and pH indicator paper. The salinity of water was determined by quantitation of Cl^−^ ions by AgNO_3_ titration (Greenberg et al. 1992) and conductivity meter. Flame photometer was used to determine the amount of Na^+^ and K^+^ ions in water sample. Tap water was used as a negative control. The concentration of Na^+^ and K^+^ ions were calculated using NaCl and KCl standard, respectively.

### Isolation of halophilic bacteria from soil sample

To isolate the micro flora from soil sample of Lunsu water body, serial dilutions (10^−1^ to 10^−5^) of enriched micro flora were spread on LB agar medium or LB agar medium supplemented with 1 M NaCl, and incubated at 30°C for 24 h. The individual colonies obtained were counted and expressed as colony forming units (CFU/g of soil) to estimate microbial load. The bacterial isolates were selected based on the size, shape, colour and texture, and purified by three successive streaking on LB agar medium containing 5.8% NaCl. Purified bacterial isolates were verified by Gram’s staining and microscopic examination. *Escherichia coli* DH5α, the laboratory strain, was used as a non-halophilic control for growth on medium supplemented with NaCl. The purified bacterial isolates were cultured and maintained on LB agar containing 5.8% NaCl.

### Biochemical characterisation of halophilic bacterial isolates

Bacterial isolates were studied for Gram’s reaction, cell morphology and pigmentation. Enzymatic assays (oxidase, catalase, nitrate reductase and urease), and assays for fermentation of lactose and mannitol were done as described by Smibert and Krieg (1994).

### Optimization of growth conditions

Different physical (pH and temperature) and nutritional (carbon and nitrogen sources) parameters were optimized for the growth of halophilic bacterial isolates. Growth parameters were studied qualitatively by streaking the bacterial isolates on LB agar medium and quantitatively by growing the bacterial isolates in LB or minimal medium (M9) at 30°C with shaking at 200 rpm for 24 h and measuring the cell density at 600 nm. To study the effect of NaCl or KCl on the growth, bacterial isolates were streaked on LB or M9 agar medium supplemented with different concentration of NaCl (0–29%) or KCl (0–18.3%). The effect of pH on the growth of halophilic bacterial isolates was studied by streaking halophilic bacterial isolates on above described growth media containing NaCl and adjusted to acidic pH of 5 and 6 by using 1N HCl and alkaline pH of 8, 9, 10, 11 and 12 using 5N NaOH^.^ The petriplates were incubated at 30°C for 24 h and observed for growth. The effect of temperature was studied by culturing the bacterial isolates in LB or M9 media at different temperature of incubation (15–50°C) for 24 h. The effect of carbon and nitrogen sources was studied by comparing the growth of isolates in M9 medium supplemented with different carbon sources (2%) such as glucose, starch, sucrose, fructose, trehalose, glycerol, lactose, raffinose, galactose, and sorbitol, and various nitrogen sources (0.25%), such as yeast extract, peptone, beef extract, casein hydrolysate, and urea respectively.

### Molecular characterization of halophilic bacterial isolates by RAPD and 16S rDNA analysis

The halophilic bacterial isolates were cultured in their optimal growth conditions to an A_600_ of ~1.0 and the cells were harvested by centrifugation at 12,000*g* for 5 min. Genomic DNA from each of the bacterial cell pellet was isolated as described by Sambrook et al. (2009).

To study the genetic relationship between the halophilic bacterial isolates, 100 ng of genomic DNA was subjected to PCR by using four different random primers named as 1K, 2K, 15K and 25K (Additional file 1: Table S1). The PCR reaction conditions were set as initial denaturation at 94°C for 2 min followed by 35 cycles of denaturation (94°C, 30 s), annealing (40°C, 30 s), and extension (72°C, 2 min), with a final extension of 10 min at 72°C. The amplified products were separated by electrophoresis on 1.2% agarose gel, stained with ethidium bromide (0.5 µg/ml) and visualised in UV gel documentation system (Alpha Innotech, USA). Towards molecular identification of halophilic bacterial isolates, 100 ng of total genomic DNA was subjected to PCR amplification of 16S rDNA gene using 27F and 1492R primers (Additional file 1: Table S1; Lane 1991). The PCR thermal cycling conditions were set as initial denaturation at 94°C for 2 min followed by 35 cycles of denaturation (94°C, 30 s), annealing (45°C, 30 s), and extension (72°C, 2 min), with a final extension of 10 min at 72°C. The PCR products were resolved on 1% agarose gel and visualised as described above. The gel purified PCR products of 16S rDNA gene were sequenced on both strands using the primers 27F and 1492R at Eurofins, Bangalore, India (https://www.eurofins.com). The nucleotide sequence thus obtained were manually analysed, overlapping sequences were removed and the complete 16S rDNA sequence for each bacterial strain was generated. Nucleotide sequences were analysed by BLAST (blastn) search and compared against bacterial 16S rDNA sequences available in the Gene bank data base (Alschul et al. 1990). The sequences were aligned by using Clustal W 1.74 (Thompson et al. 1994), followed by construction of neighbour joining phylogenetic tree using MEGA4 (http://www.megasoftware.net) (Tamura et al. 2007). The nucleotide sequences were submitted in the GenBank data base (https://www.ncbi.nlm.nih.gov/genbank/‎).

## Results

### NaCl is the predominant salt in Lunsu water

Lunsu is a saltern water body that originates from the Rocky Mountains in the foot hills of Himalaya (Additional file 2: Figure S1). At the time of sample collection, the pH of water sample was slightly acidic (pH 6), and the temperature was 21°C at noon time. The water sample tested positive for the presence of salts by conductivity tests. Volumetric analysis revealed that the concentration of sodium chloride was 1.39%. By using flame photometric assay, it was found that Lunsu water contains 500 times more sodium ions (1.39%) as compared to potassium ions (0.0035%).

### Halophilic and halotolerant bacteria are present in the soil sediments of Lunsu water body

To explore the presence of halophiles in the Lunsu water body, soil sediments were spread on bacterial growth medium in the absence and presence of NaCl. Microbial growth was observed in both the media, with the microbial load of 4 × 10^5^ CFU/g of soil on LB, and 3 × 10^4^ CFU/g of soil on LB medium supplemented with 1 M NaCl. Colonies of different colours (yellow, orange, creamish and white) were observed. Very interestingly, yellow/orange pigmented colonies showed growth only on medium supplemented with NaCl (data not shown). This indicates that yellow/orange pigmented colonies are strict halophiles, which require NaCl for growth; whereas white/creamish coloured colonies do not require NaCl for growth. This data is in agreement with the fact that the soil sediment of Lunsu water body naturally appears orange in colour (Additional file 2: Figure S1).

### Morphological and biochemical characteristic features of halophilic bacterial isolates

**Figure 1:**
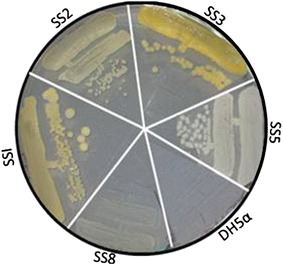
Growth of halophilic bacterial isolates. Purified bacterial strains as indicated were streaked on LB agar medium supplemented with 1 M NaCl and incubated for 48 h at 37°C. *E. coli* strain DH5α was used as non-halophilic control.

A total of five distinct bacterial isolates (SS1, SS2, SS3, SS5 and SS8) were isolated from soil sediments of Lunsu (Figure 1). The colour of the isolates varied from creamish, whitish, pale yellow to orange (Table 1). The bacterial isolates SS1, SS2, SS3 and SS5 were motile, rod shaped and catalase positive, where as SS8 was cocci shaped, catalase and oxidase negative (Table 1). Bacterial isolates SS1 and SS3 were positive for mannitol fermentation and negative for oxidase and nitrate tests. Urease activity was observed in all the bacterial isolates, except SS1. All the bacterial isolates were negative for lactose fermentation.

**Table 1:** Biochemical and growth characteristics of the halophilic bacterial isolates of Lunsu

Cellular/biochemical and growth parameter			Halophilic bacterial isolate				
			SS1	SS2	SS3	SS5	SS8
A	Cell morphology		Rods	Rods	Rods	Rods	Cocci
B	Motility		+	+	+	+	+
C	Pigmentation		Pale yellow	Whitish	Yellowish	Whitish	Creamish
D	Gram’s reaction		+	–	+	–	–
E	Catalase		+	+	+	+	–
F	Oxidase		–	+	–	+	+
G	Nitrate		–	+	–	–	+
H	Urease		–	+	+	+	+
I	Mannitol fermentation		+	_	+	_	_
J	NaCl (%) optimum concentration (range)		11.6 (3.8–26.1)	4.3 (0–8.7)	11.6 (3.8–26.1)	8.7 (14.5)	2.9 (0–8.7)
K	KCl (%) optimum concentration (range)		_	3.7 (0–7.4)	_	7.4 (0–14.8)	3.7 (0–7.4)
L	pH for growth (optimum pH (range)		9 (7–12)	7 (6–12)	9 (7–12)	8 (6–12)	7 (6–8)
M	Temperature (°C) optimum temperature (range)		37 (25–40)	37 (25–40)	37 (25–40)	37 (15–40)	30 (15–37)
N	Glucose	Yeast extract	–	–	–	–	–
O	Glucose	Beef extract	+	+	–	+	+
P	Glucose	Peptone	+	+	–	+	+
Q	Glucose	Casein hydrolysate	+	+	+	+	+
R	Raffinose	Casein hydrolysate	–	–	–	+	–
S	Lactose	Casein hydrolysate	–	–	–	+	–
T	Trehalose	Casein hydrolysate	–	–	–	+	–
U	Starch	Casein hydrolysate	–	–	–	+	–
V	Galactose	Casein hydrolysate	–	–	–	+	–
W	Fructose	Casein hydrolysate	–	–	–	+	–
X	Sucrose	Casein hydrolysate	–	–	–	+	–
Y	Sorbitol	Casein hydrolysate	–	–	–	+	–
Z	Glycerol	Casein hydrolysate	–	–	–	+	–

Plus (+) sign indicates the test as positive, where as negative (−) sign indicates negative results for biochemical test. Plus (+) sign indicates the growth of bacterial isolate and negative (−) sign indicates no detectable growth when streaked or spotted on LB/M9 agar medium.

### SS1 and SS3 are strict halophiles while SS2, SS5 and SS8 are halotolerant bacteria

We isolated halophilic bacteria on growth medium with or without NaCl supplementation at pH 7 and temperature of 30°C. Moreover, we detected both Na^+^ and K^+^ ions in the water sample. Therefore, we studied the effect of NaCl, KCl, temperature and pH on the growth of bacterial isolates. Out of the five bacterial isolates, SS1 and SS3 did not show any growth on LB containing less than 3.8% NaCl (Table 1) and exhibited growth up to 26.1% NaCl, thus indicating the strictly halophilic nature of the isolates. However, no detectable growth of SS1 and SS3 was observed in the presence of ≥0.74% KCl (Table 1).

The bacterial isolates SS2 and SS8 exhibited growth even in absence of NaCl or KCl and tolerated up to 8.7% NaCl or 7.4% KCl (Table 1). The bacterial isolate SS5 showed growth in absence or presence of NaCl or KCl, and growth was observed up to 14.5% NaCl or 14.8% KCl. Based on these observations, we conclude that bacterial isolates SS2, SS5 and SS8 are halotolerant bacteria.

The optimum growth of isolates SS1 and SS3 was observed in the presence of 11.6% NaCl, whereas it was 8.7, 4.3 and 2.9% NaCl for SS5, SS2 and SS8 respectively. While SS1 and SS3 did not show any growth in the presence of KCl, SS5 showed optimum growth in the presence of 7.4% KCl and SS2 and SS8 in the presence of 3.7% KCl (Table 1).

### SS1, SS3 and SS5 are haloalkaliphilic while SS2 and SS8 are haloneutrophilic in nature

The halophilic isolates SS1 and SS3 showed growth in LB medium supplemented with 11.6% NaCl and pH range of 7–12. The optimum pH for the growth of SS1 and SS3 was 9 and they did not show any detectable growth at or below pH 6 (Table 1), which indicates the alkaliphilic nature of SS1 and SS3 isolates.

The bacterial isolates SS2 and SS5 exhibited growth in the range of pH 6–12, while SS8 did not show any growth at pH greater than 8. The optimum pH for growth of strain SS5 was pH 8.0, whereas for SS2 and SS8 was pH 7.0 (Table 1). These results indicate that SS1, SS3 and SS5 are alkaliphilic, while SS2 and SS8 are neutrophilic in nature.

Two halophiles (SS1 and SS3) and two halotolerants (SS2 and SS5) showed growth between 25 and 40°C in LB medium supplemented with 11.6 and 5.8% NaCl respectively, with an optimum growth at 37°C (Table 1). The bacterial isolate SS8 exhibited optimal growth at 30°C, poor growth at 37°C and no detectable growth at 40°C. Also, detectable growth of halophilic isolates SS5 and SS8 was observed at 15°C after incubation of 72 h. However, none of the bacterial strains could grow below 15°C and above 45°C (Table 1).

### The halophilic and halotolerant bacterial isolates exhibit complex growth requirement

To characterise the growth in minimal and defined medium, we studied the effect of different carbon and nitrogen sources on the growth of halophilic bacterial isolates. It was observed that Minimal medium (M9) containing glucose as a carbon source and ammonium chloride as a nitrogen source did not support growth of any of the halophilic isolates (Table 1). More importantly, growth was also not observed when ammonium chloride was replaced with urea or yeast extract as a nitrogen source. The addition of casein hydrolysate as a nitrogen source supported the growth of all the halophilic isolates (Table 1). Since glucose as carbon source and casein hydrolysate as a nitrogen source supported the growth of all the bacterial isolates, we studied the effect of different carbon sources in M9 medium supplemented with casein hydrolysate as nitrogen source. Very surprisingly, none of the bacterial isolates, except SS5, showed growth when glucose, starch, sucrose, fructose, trehalose, glycerol, lactose, raffinose, galactose or sorbitol was supplemented as carbon source (Table 1). All halophilic isolates showed growth in presence of peptone and beef extract except isolate SS3. (Table 1). Together, these results indicate a complex requirement of carbon and nitrogen sources by the halophilic bacterial isolates of Lunsu. The effect of temperature, pH and NaCl concentrations were also studied in M9 medium supplemented with casein hydrolysate and glucose (data not shown). There was no significant difference in the optimum pH, temperature or salt requirement as compared to those in LB medium (Table 1).

### Halophilic bacterial isolates of Lunsu are phylogenetically different

Each of the five halophilic bacterial isolate (SS1, SS2, SS3, SS5 and SS8) showed distinct morphological and physiological characteristic features. In order to confirm their distinctness at the molecular level, RAPD analysis was performed using a set of four random primers (Additional file 1: Table S1). The total genomic DNA of halophilic bacterial strains was isolated (Additional file 3: Figure S2a) and subjected to RAPD amplification using random decamer primers. RAPD analysis revealed differences in the number and banding pattern of each of the five isolates (Additional file 3: Figure S2b–e). In general, the size of DNA amplification products varied from 0.1 to 5 kb. The primer 1K generated PCR products ranging 0.25–3.2 kb (Additional file 3: Figure S2b), primer 2K between 0.75 and 3 kb (Additional file 3: Figure S2c), primer 15K between 0.1 and 4.8 kb (Additional file 3: Figure 2Sd) and primer 25 K between 0.2 and 5 kb (Additional file 3: Figure 2Se). The largest number of polymorphic bands was observed with primer 15K, followed by 1K, 25K and 2K. Thus, RAPD analysis revealed that all the five halophilic isolates are genetically distinct, with close relatedness in SS1and SS3, SS2 and SS5, while SS8 being a divergent member.

### Molecular identification of the halophilic bacterial isolates

In order to identify the halophilic bacterial isolates, 16S rDNA gene was amplified using gene specific primers. A PCR product of ≈1.5 kb was detected in all the five isolates. The 16S rDNA amplicons of each bacterial isolate was sequenced on both strands using 27F and 1492R primers. The complete nucleotide sequence of 1,461, 1,412, 1,437,1,408 and 1,416 bp sequences were obtained from SS1, SS2, SS3, SS5 and SS8 isolates respectively, and subjected to BLAST analysis. 16S rDNA sequence analysis showed that the isolated strains belong to the genera *Halobacillus*, *Shewanella*, *Halomonas* and *Marinomonas*. The halophilic isolates SS1 and SS3 showed 98 and 99% similarity, respectively with *Halobacillustrueperi* DSM10404 (accession no. NR_025459.1). The isolates SS5 and SS8 showed 98% similarity with *Halomonas venusta* DSM4743 (accession no. NR_042069.1) and *Marinomonas* sp. BSi20328 (accession no. NR_043882.1), respectively. The bacterial isolate SS2 showed 99% sequence similarity with *Shewanella algaeOK*-*1* (accession no. NR_028673.1). The 16S rDNA nucleotide sequences of all the five halophilic bacterial strains have been submitted to the NCBI GenBank database under the accession nos. KM260166 (*Halobacillustrueperi* strain SS1), KF751760 (*Shewanella algae* strain SS2), KF751761 (*Halobacillustrueperi* strain SS3*)*, KF751762 (*Halomonas venusta* strain SS5) and KF751763 (*Marinomonas* strain SS8).

**Figure 2:**
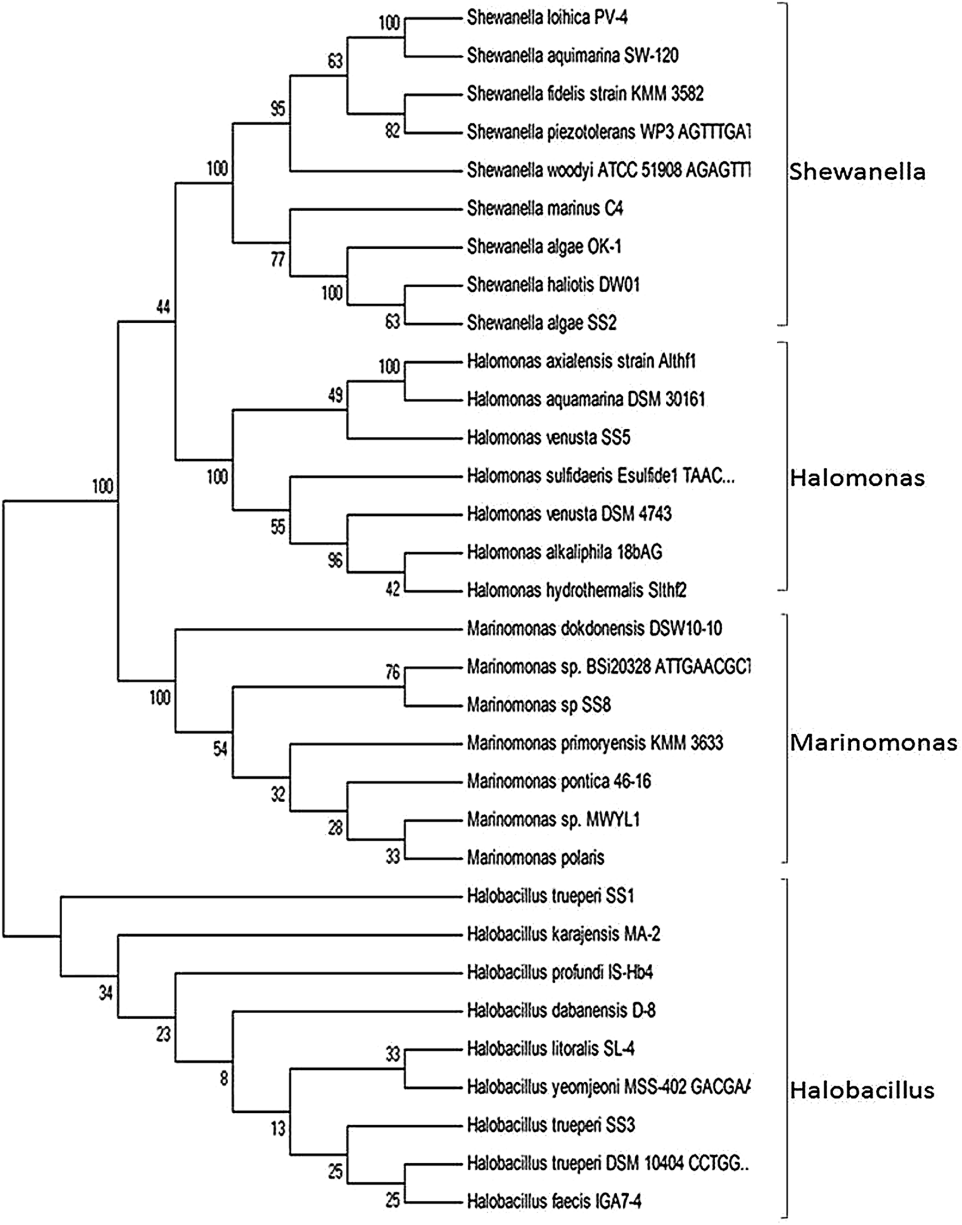
Phylogenetic analysis of halophilic bacterial isolates based on 16S rDNA sequences. Neighbour-joining phylogenetic trees depicting the interrelationships of 16S rDNA sequence of halophilic isolates SS1, SS3, SS2, SS5 and SS8 with closely related halophilic isolates of their respective genera.

To validate the molecular identity of the five halophilic bacterial strains of Lunsu, phylogenetic analysis of 16S rDNA was carried out for all the five halophilic bacterial strains along with the isolates showing more than 90% similarity. The halophilic bacterial isolates formed five independent clusters and showed genetic relatedness to the members of their respective genera (Figure 2). *Halobacillustrueperi* SS3 evolutionarily evolved with *Halobacillustrueperi* DSM10404 and *Halobacillus faecis* IGA7-4. On the other hand, *Halobacillustrueperi* SS1 formed a separate clad from the other members of the *Halobacillus* spp. It is also important to note that *Halobacillustrueperi* strain SS1 and *Halobacillustrueperi* strain SS3 fall into separate phylogenetic clads, despite living in a common habitat. *Shewanella algae* SS2 clustered with *Shewanella haliotis DW01*, despite of its 99% sequence similarity with *Shewanella algaeOK*-*1* (accession no. NR_028673.1). *Halomonas venusta* strain SS5 formed a cluster with *Halomonasaquamarina* DSM30161 and *H. axialensis* despite its similarity (99%) with *Halomonas venusta* DSM4743. *Marinomonas* strain SS8 co-evolved with *Marinomonas* sp. BSi20328 (Figure 2).

## Discussion

Recent decades have seen a rise in studies on microorganisms isolated from extreme environments, including hyper saline ecosystems. Both molecular and microbiological studies have revealed the existence of moderate to extremely halophilic micro and some macro-organisms in a wide range of these saline environments (Ventosa et al. 1998; Roohi et al. 2012; Hedi et al. 2014**)**. In the present study, five bacterial strains have been isolated from the soil sediments of Lunsu water body of Himachal Pradesh, India.

The present study showed that Lunsu water sample contains 1.38% Na^+^ ions (1.38% NaCl) and 0.0035% K^+^ ions (0.0035% KCl), which is 24 times more than the amount of Na^+^ ions in tap water. The high concentration of NaCl in the water was consistent with the fact that the water of Lunsu has an unbearable salty taste (observations of the local people). The salt content estimates indicate that the Lunsu water body represents a moderately hyper saline environment. Several hyper saline environments have been studied worldwide, of which Dead Sea has the maximum salt content (DasSarma and Arora 2001; Grant 2004). It has been reported that the concentration of NaCl and KCl in Dead Sea ranges from 7.1 to 9.1% and 1.5 to 1.7%, respectively (Wisniak 2002). Amongst the hypersaline habitats in India, the Lonar Lake in Maharashtra has been reported to contain 0.35% Na^+^ and 0.0017% K^+^ ions (Pedge and Ahirrao 2013).

The halophilic bacterial isolates SS1 and SS3 showed growth at temperature of 25–40°C, pH 7.0–12.0 and 3.8–26.1% NaCl. Bacterial isolate SS5 showed detectable growth at the temperature of 15–40°C, pH 6.0–12.0 and in the presence of 0–14.5% NaCl whereas SS8 showed growth at temperature of 15–37°C, pH 6.0–8.0 and in the presence of 0–8.7% NaCl. The growth of bacterial isolate SS2 was observed in the presence of 0–8.7% NaCl, pH 7.0–12.0 and temperature of 25–40°C. Kanekar et al. (2008) isolated Gram’s positive bacterial strains from Lonar Lake, which showed optimum growth at 23°C, pH 9.8 and 4.5% NaCl. Vidyasagar et al. (2007) isolated gram negative, rod shaped halophilic strains from solar evaporation pond with optimum growth at 23% (w/v) NaCl. Patel et al. (2010) have reported halophilic gram-negative strains from the Arabian coasts, Gujarat, India. However, there are no reports on halophiles from Northern India, including Himachal Pradesh. Thus, our study forms the first report on the characterization of halophiles from Northern India.

The molecular identification of the halophiles from Lunsu revealed that the SS1 and SS3, are ≥98% identical *Halobacillustrueperi* and SS2, SS5 and SS8 are identical to *Shewanella algae*, *Halomonas venusta* and *Marinomonas* sp. respectively. Spring et al. (1996) described the isolation of orange pigmented *Halobacillus trueperi* from Great Salt Lake in Utah, which exhibited growth at temperature range of 10–44°C and pH 6.0–9.5 in the presence of 0.5–30% (w/v) NaCl. Recently, rod shaped, creamish pigmented *Halobacillus* sp. has been reported from Sehline Sebkha salt lake, Tunisia, which showed growth in presence of 5–25% (w/v) NaCl at 37°C (Hedi et al. 2014). Similarly, halophilic bacterial isolates *Halobacillus trueperi* SS1 and SS3 of Lunsu have yellow–orange pigmentation and exhibit growth in the presence of 4–26.1% (w/v) NaCl, pH 7–12 and temperature 25–40°C. In addition, *Halobacillus trueperi* SS1 and SS3 failed to grow in presence of KCl and below 3.8% NaCl. No such reports exist about *Halobacillus trueperi* from other studies. Together, our study indicates that *Halobacillus trueperi* SS1 and SS3 exhibit significant differences in their growth characteristics from known species of this genus.

Generally, *Halomonas* sp. are creamish to whitish in colour (Mata et al. 2002), except that *Halomonas venusta* ATCC 27125 was found to be yellow in colour and exhibited growth in the presence of 0–20% (w/v) NaCl, pH 5–10 and temperature ranging from 4 to 45°C (Mata et al. 2002). The halophilic bacterial strain SS5 of Lunsu has 98% identity with *Halomonas venusta* DSM 4743. *Halomonas venusta* SS5 showed similar properties (Table 1) with exception to the whitish colour as reported by Mata et al. 2002.

The halo bacterial strain SS2 of Lunsu exhibited 99% sequence similarity to *Shewanella algaeOK*-*1.* Most of the species of these genera have been isolated from marine regions and thus consistent of being halotolerant in nature. For example, *Shewanella haliotis DW01T* was isolated from the gut micro flora of abalone collected from the South Sea (Kim et al. 2007). Another bacterial strain *Shewanella algae ATCC 51192* was isolated from salt marshes that formed pink colour colonies and showed salt tolerance up to 6% (w/v) NaCl (Nozue et al. 1992). On the other hand *Shewanella algae* SS2 isolated in the present study is whitish in colour and exhibited growth in the presence of 0–8.7% (w/v) NaCl.

Recently, *Halomonas* sp. and *Shewanella* sp. were also reported from the foreshore soil of Daecheon beach and Saemangeum sea of Korea (Irshad et al. 2014). As the name suggests, members of *Marinomonas* have been isolated from marine and coastal regions (Dong et al. 2014). It is interesting to note that halophiles found in marine areas (*Shewanella* sp. and *Marinomonas* sp*.)* also inhabit the hilly saline water of Lunsu. *Marinomonas* strain IVIA-Po-185T and *M. pontica* 46-16 have been isolated from the sea grass *Posidonia oceanic*. They are helical in shape, have salt tolerance up to 10% and grow at temperatures between 5–37°C and 4–33°C, respectively (Espinosa et al. 2010). The halotolerant isolate *Marinomonas* sp. SS8 of Lunsu showed similar features including growth temperature of 15–37°C and salt tolerance up to 8.7% (w/v) NaCl.

## Conclusion

To our knowledge, this is the first study on the microbial diversity of halophilic bacterial community inhabiting the saltern water body. Two types of halophilic and halotolerant bacteria have been isolated from soil sediment of Lunsu, which are phylogeneticaly different. Culturing of microbes and their molecular analysis provides an opportunity to have a wide range of cultured microorganisms from the extreme environments. The microbial diversity of extremophiles can prove to be a valuable resource in various industrial and biotechnological processes requiring specialised features. Halophiles offer an added advantage to be a source of gene(s) that can increase salt tolerance in different crops through genetic engineering techniques. Thus, the halophiles isolated from Lunsu water body offer an important potential for application in microbial, enzyme and agricultural biotechnology.

## Additional files

**Additional file 1: Table S1.** Primers used in the present study. 
**Additional file 2: Figure S1.** Source of halophilic microbial isolates. A picture of Lunsu water body in Lunsu village, Kangra district, Himachal Pradesh, India. The location of sample collection is indicated.
**Additional file 3: Figure S2.** RAPD- PCR analysis of halophilic microbial isolates. (a) Genomic DNA of halophilic bacterial strains was isolated and electrophoresed on 1% agarose gel. Lane M indicates the DNA molecular size marker (kb) and genomic DNA in other lanes as indicated. b-e: PCR amplified products using random primers 1K (b), 2K (c), 15K (d) and 25K (e) were resolved on 1.2% agarose gel. Lane M indicates molecular size marker (kb) and remaining lanes contained RAPD products of different halophilic bacterial isolates as indicated.
