# Supplementary material for: A diverse group of halophilic bacteria exist in Lunsu, a natural salt water body of Himachal Pradesh, India
Source: Springerplus. 2015 Jun 17;4:274. doi: 10.1186/s40064-015-1028-1 (PMC4469599; doi:10.1186/s40064-015-1028-1)
Supplement: Supplementary file 1 — Additional file 1: Table S1. Primers used in the present study. [file 40064_2015_1028_MOESM1_ESM.doc]

| **Primer**  **Name** | **Sequence (5’-3’)** | **Length (nucleotides)** | **Tm (°C)** | **Reference** |
| --- | --- | --- | --- | --- |
| 27F | AGAGTTTGATCTGGCTCAG | 19 | 45 | Lane, 1991 |
| 1492R | ACCTTGTTACGACTT | 15 | 45 | Lane, 1991 |
| 1K | CAGGCCCTTC | 10 | 40 | This study |
| 2K | TGCCGAGCTG | 10 | 40 | This study |
| 25K | GTCGCCGTCA | 10 | 40 | This study |
| 15K | GATGACCGCC | 10 | 40 | This study |
