# Supplementary figures and images for: A diverse group of halophilic bacteria exist in Lunsu, a natural salt water body of Himachal Pradesh, India
Source: Springerplus. 2015 Jun 17;4:274. doi: 10.1186/s40064-015-1028-1 (PMC4469599; doi:10.1186/s40064-015-1028-1)

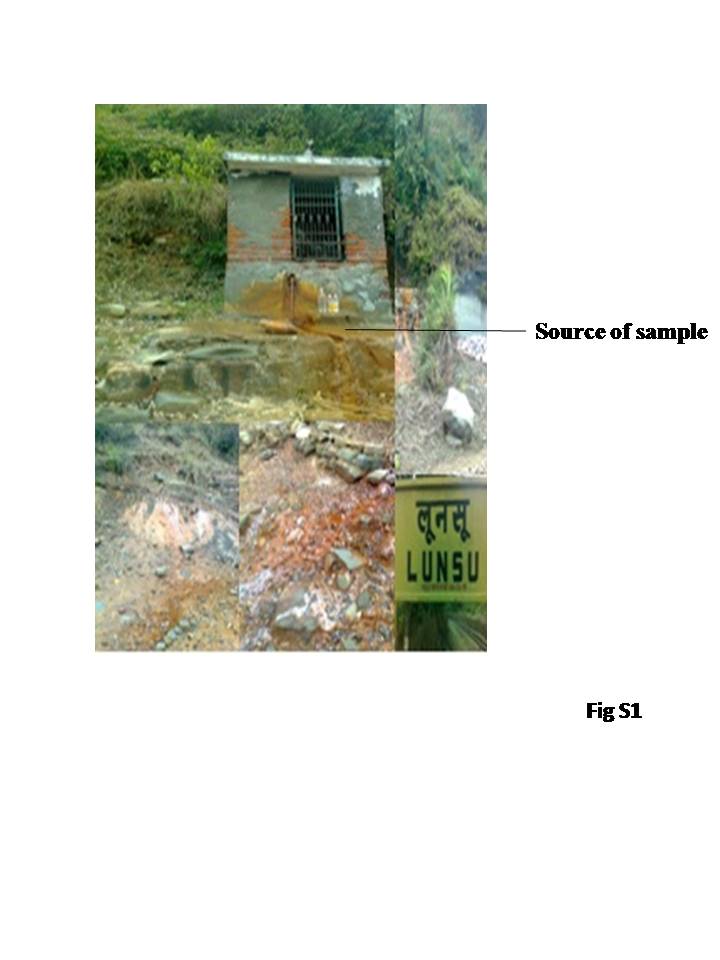

Supplement: Supplementary file 2 — Additional file 2: Figure S1. Source of halophilic microbial isolates. A picture of Lunsu water body in Lunsu village, Kangra district, Himachal Pradesh, India. The location of sample collection is indicated. [file 40064_2015_1028_MOESM2_ESM.jpeg]

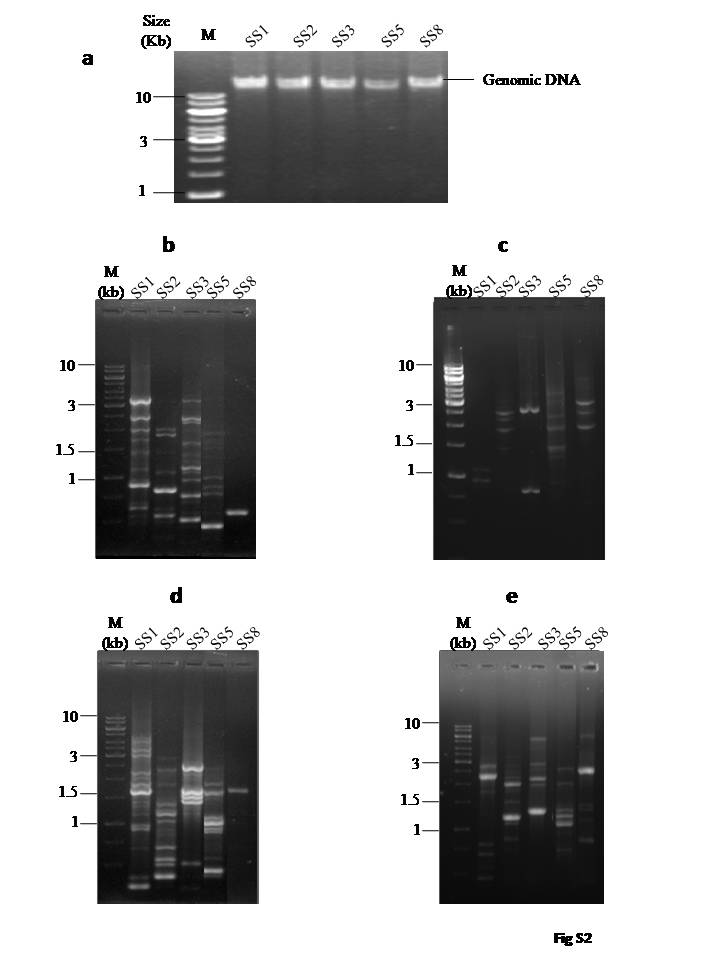

Supplement: Supplementary file 3 — Additional file 3: Figure S2. RAPD- PCR analysis of halophilic microbial isolates. (a) Genomic DNA of halophilic bacterial strains was isolated and electrophoresed on 1% agarose gel. Lane M indicates the DNA molecular size marker (kb) and genomic DNA in other lanes as indicated. b-e: PCR amplified products using random primers 1K (b), 2K (c), 15K (d) and 25K (e) were resolved on 1.2% agarose gel. Lane M indicates molecular size marker (kb) and remaining lanes contained RAPD products of different halophilic bacterial isolates as indicated. [file 40064_2015_1028_MOESM3_ESM.jpeg]
